# Negative central venous to arterial lactate gradient in patients receiving vasopressors is associated with higher ICU 30-day mortality: a retrospective cohort study

**DOI:** 10.1186/s12871-021-01237-5

**Published:** 2021-01-22

**Authors:** Qing Zhang, Ye Liu, Longxiang Su, Wenzhao Chai, Hongmin Zhang, Xiaoting Wang, Dawei Liu

**Affiliations:** 1 Department of Critical Care Medicine, Peking Union Medical College Hospital, Peking Union Medical College & Chinese Academy of Medical Sciences, Shuaifuyuan, Wangfujing, Dongcheng district, Beijing, 100730 China; 2 Department of Health Care Organization and Policy, School of Public Health, University of Alabama at Birmingham, 1665 University Boulevard, Birmingham, AL 35294-0022 USA

**Keywords:** Hemodynamic monitoring, Hyperlactatemia, Mortality

## Abstract

**Background:**

Serum lactate has long been used to evaluate hypoxia and predict prognosis in critically ill patients, however, discrepancy in lactate measurements between different sites have not been recognized as a useful tool for monitoring hypoxia and evaluating outcome.

**Methods:**

Data were obtained from the clinical information system of the intensive care unit (ICU) in a tertiary academic hospital for 1582 ICU patients with vasoactive drug requirement and valid paired blood gas. The mortality rates were compared between patients with sustained negative venous to arterial lactate gradient (VALac) and the others using the Cox proportional hazard model. Predictive factors associated with negative VALac were searched.

**Results:**

A sustained negative VALac was significantly associated with higher 30 day ICU mortality [Adjusted hazard ratio (HR) = 2.31, 95% confidence interval (CI), 1.07–4.99; *p* = 0.032. Propensity score- weighted HR: 2.57; 95% CI, 1.17–5.64; *p* = 0.010]. Arterial lactate in the first blood gas pair, 24-h arterial lactate clearance, use of epinephrine, mean positive end-expiratory pressure level, and extracorporeal membrane oxygenation initiation showed statistically significant association with sustained negative VALac during the first 24 h.

**Conclusion:**

The sustained negative VALac in the early stage of treatment may suggest additional information about tissue hypoxia than arterial lactate alone. Critical care physicians should pay more attention to the lactate discrepancy between different sites in their clinical practice.

**Supplementary Information:**

The online version contains supplementary material available at 10.1186/s12871-021-01237-5.

## Background

Tissue hypoxia is one of the main causes of multiple organ dysfunction syndrome in circulatory shock. Systemic markers measured by blood gas analysis, including lactate, have long been used in clinical practice for diagnosing and evaluating tissue hypoxia in critically ill patients of various causes [1–5]. Unlike the arterial lactate, lactates from peripheral or central veins, right atrium, or pulmonary artery were not routinely used for the clinical purpose and sometimes were tested as surrogates for arterial lactate [6]. However, lactate levels from different sites do not always converge [7]. The discrepancy may reflect the pathological status. Kellum et al. [8] showed that in patients with acute lung injury and hyperlactatemia the lung was a major source of lactate. The lactate level has also been shown to be lower in the coronary vein [9, 10]. As described by Jalloh et al. [11], the mean arterial-jugular bulb lactate differences were positive in patients with brain injury but were negative in normal controls, which suggested the uptake of lactate in the injured brain. Difference in lactate metabolism in various pathophysiological statuses may contribute to the discrepancy in lactate levels between different sites, such as artery and central vein, and may provide additional information on oxygen metabolism which may not be reflected by arterial lactate only. Evidence focusing on the lactate discrepancy and patients’ outcome are limited. We hypothesize that a sustained negative central venous to arterial lactate gradient is associated with a poor outcome in patients receiving vasopressors.

## Methods

### Setting and data source

This retrospective cohort study was conducted at a general intensive care unit (ICU) in a large tertiary academic hospital in Beijing, China. Patients were admitted to our ICU due to both post-surgical and medical reasons, including some high-risk post-anesthesia care patients. We used the data from the Critical Care Information System (CCIS, DHC Software, Co., Ltd. Beijing, China) of our department, which is a point-of-care information system that automatically collects patients’ information during routine care from bedside devices and electronic medical records. This database has been previously used to evaluate the prognosis and treatments of critically ill patients in our department [12]. This study was approved by the Institutional Research and Ethics Committee of our hospital. Informed consent was waived based on the study’s retrospective, observational design, which preserved the confidentiality of personal information.

### Study cohort

We used the data from Jan.1st, 2016 to Jun. 30th, 2018. During this period, all patients admitted to our ICU who were treated for no less than 24 h and received vasopressors and/or inotropes within the first 24 h after admission were included. The paired blood gas tests were requested by physicians when they needed more information about patient’s hemodynamic status. In this study, paired blood gas was defined as 1 arterial and 1 central venous blood gas (obtained from superior vena cava (SVC) via central venous catheter, the position of which was confirmed by the bedside chest X-ray) results obtained within 20 min. Patients who had at least 1 paired blood gas result were selected. We excluded patients who were younger than 18 years old or had incomplete baseline characteristics. Patient can be enrolled only once.

For the primary analysis, we only included patients whose first blood gas pairs were obtained within the first 8 h after admission and had at least 2 valid blood gas pairs during the first 24 h. Bedside blood gas machines used in our department were GEM Premier 3000, model 5700 (Lexington, MA, USA) and ABL90, Radiometer (Copenhagen, Denmark), during the study period.

### Exposure and outcome

We used the central venous to arterial lactate gradient (VALac = central venous lactate – arterial lactate) to define our exposure factor. We calculated all VALac and counted the number of differences that were negative within the first 24 h after admission for each patient. We defined the primary exposure factor, i.e., the sustained negative VALac, as the negative VALac appeared in more than 50% of all results within the first 24 h. The study cohort was therefore divided into 2 groups, i.e., the sustained negative VALac group (exposure group) and the control group. The primary outcome was the ICU 30-day mortality.

Follow-up began on the second day of ICU stay and ended if the patient was discharged, dead, or at the 30-day follow-up period, whichever happened first.

### Covariates

Covariates were identified and extracted from CCIS. We selected demographic characteristics, baseline comorbidities, acute scenarios and severity scores at admission, and treatment information within the first 24 h as covariates. We used the number of blood gas pairs as the proxy for monitoring and treatment intensity. Detailed covariates were listed in the Supplementary data.

### Statistical analysis

Continuous variables were presented as medians and interquartile ranges (IQR). Categorical variables were presented as frequencies with percentages. To compare the baseline characteristics, the Mann- Whitney U test was used for the continuous variables, and the chi-square test or Fischer exact test were used for categorical variables where appropriate.

In our primary analysis, the Kaplan-Meier plot and the log-rank test were used to compare the survival distributions between two groups. Continuous variables were explored for monotonicity and linearity of the association with mortality before using in the models. The adjusted hazard ratio (HR) with 95% confidence interval (CI) was obtained by a multivariable Cox proportional hazard model accounting for potential confounders. The effect of including each potential covariate in a Cox model was calculated once at a time. Those covariates influencing the HR by more than 5% were then included in multivariable Cox models, and the backward elimination was performed using the Wald test.

To better balance the baseline characteristics, a propensity score (PS)-weighting analysis was used as secondary analysis. We used the matching weight method developed by Li and Greene [13], which has been recently used by Sauer et al. [14] . Propensity scores of exposed individual were estimated by a multivariable logistic regression model using all covariates mentioned above. The balance of baseline characteristics of the weighted cohort was tested by standard mean difference (SMD) of each variable. Kaplan-Meier curve and a univariable Cox model were then performed in the weighted cohort to evaluate the survival impact.

To further evaluate the association between sustained VALac and the 30-day mortality, we repeated the primary analysis using patients whose all VALac within the first 24 h were negative as exposure group.

To investigate the relationship between covariates and our primary exposure factor, univariable and multivariable logistic regression models were used to test the association between the sustained negative VALac and the potential covariates. The covariates that showed *p* value < 0.2 in univariable models were included in the multivariable model. Odds ratios (OR) with 95% CI were reported.

### Sensitivity analysis

In order to test the robustness of our results, we performed the sensitivity analyses for the primary analysis, including 1) using ICU all-cause mortality as outcome; 2) change inclusion criteria to include only patients whose first blood gas pair was obtained within 4 h after admission; and 3) change inclusion criteria to include only patients who had at least 4 paired blood gas results obtained. We used the definition of exposure same as the primary analysis.

Data were analyzed using Stata 15.1 (Stata Corp, College Station, Texas, USA). A *p*-value< 0.05 was considered as statistically significant. And all results were reported based on Strengthening the Reporting of Observational Studies in Epidemiology (STROBE) guidelines [15].

## Results

**Fig. 1:**
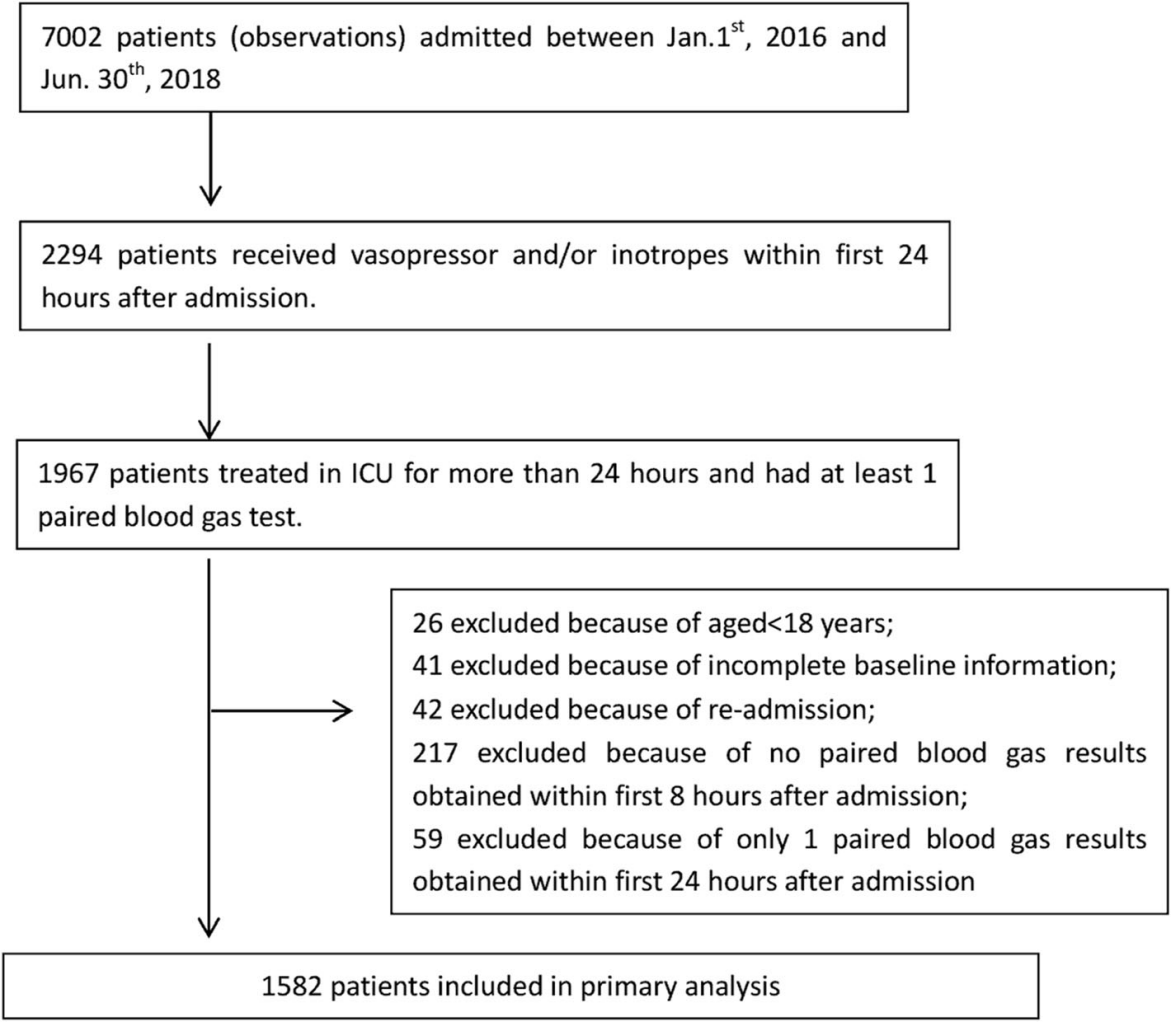
Study flow diagram

A total of 7002 patients were admitted to our ICU during the study period, among whom 2294 (32.8%) received vasoactive agents within the first 24 h after admission. The primary analysis included 1582 patients (Fig. 1). The median length of ICU stay was 5 days (IQR: 2 days, 9 days). Fifty- three patients died within 30 days in ICU (3.35%), among whom 22 were in the exposure group. The baseline characteristics and the treatment information within the first 24 h are shown in Table 1. Of note, patients who had sustained lower central venous lactate than arterial lactate had higher proportions of acute brain injury and cirrhosis. Their mean oxygen index was lower while mean positive end-expiratory pressure (PEEP) level was higher during the first 24 h in ICU, indicating more severe lung injury.

**Table 1:** Baseline and Treatment Main Characteristics of Study Cohort by Exposure Status

Characteristics	Control (***n*** = 1435)	Exposure ^a^ (***n*** = 147)	***P***-value
Age, median (IQR)	62 (50, 70)	60 (46, 68)	0.17
Female, N (%)	666 (46.4%)	67 (45.6%)	0.85
Acute brain injury, N (%)	65 (4.5%)	13 (8.8%)	0.021
Trauma or major bleeding^b^, N (%)	45 (3.1%)	7 (4.8%)	0.29
Pulmonary hypertension or PE, N (%)	66 (4.6%)	5 (3.4%)	0.50
Infection, N (%)	497 (34.6%)	54 (36.7%)	0.61
Cardiac dysfunction^c^, N (%)	312 (21.7%)	40 (27.2%)	0.13
Chronic kidney disease, N (%)	47 (3.3%)	4 (2.7%)	0.72
Cirrhosis, N (%)	16 (1.1%)	5 (3.4%)	0.039
COPD or asthma, N (%)	29 (2.0%)	2 (1.4%)	1.00
Post-anesthesia, N (%)^d^	983 (68.5%)	103 (70.1%)	0.70
APACHEII, median (IQR)	16 (12, 21)	19 (14, 28)	< 0.001
SOFA Total, median (IQR)	10 (8, 12)	12 (9, 16)	< 0.001
Arterial lactate in first blood gas pair (mmol/L), median (IQR)	2.2 (1.4, 4.2)	5.9 (3.8, 9)	< 0.001
S_cv_O_2_ in first blood gas (%), median (IQR)	74.4 (67.5, 80.8)	74.1 (67.5, 81.7)	0.72
P_cv-a_CO_2_ in first blood gas pair (mmHg), median (IQR)	5.6 (3.8, 7.6)	5.5 (3.7, 7.4)	0.79
P_cv − a_CO_2_/C_a − cv_O_2_ ratio in first blood gas pair (mmHg/ml), median (IQR)	1.49 (1.14, 1.88)	1.50 (1.18, 2.04)	0.33
Serum creatinine (μmol/L), median (IQR)	75 (59, 103)	89 (65, 138)	< 0.001
Arterial Hgb (g/L), median (IQR)	114 (98, 131)	112 (97, 127)	0.60
Treatment information within first 24 h			
Total fluid balance (ml), median (IQR)	−61.6 (− 1330.7, 1013.9)	385.6 (− 1225.8, 2628.6)	< 0.001
Transfusion	213 (19.5%)	41 (28.9%)	0.010
Albumin infusion	753 (69.0%)	100 (70.4%)	0.73
Epinephrine use, N (%)	328 (22.9%)	47 (32.0%)	0.013
Dobutamine use, N (%)	52 (3.6%)	14 (9.5%)	< 0.01
Norepinephrine use, N (%)	1395 (97.2%)	146 (99.3%)	0.13
CVVH initiation, N (%)	140 (9.8%)	45 (30.6%)	< 0.001
ECMO initiation, N (%)	5 (0.3%)	8 (5.4%)	< 0.001
PiCCO initiation, N (%)	146 (10.2%)	43 (29.3%)	< 0.001
24-h lactate clearance^e^ (%), median (IQR)	33.3 (0, 60.0)	43.1 (−5.3, 62.8)	0.56
Mean oxygen index (mmHg), median (IQR)	324.26 (257.38, 399.78)	295.42 (192.57, 382.66)	< 0.001
Mean PEEP level during first 24 h (mmHg), median (IQR)	5.0 (5.0, 5.9)	5.6 (5.0, 7.7)	< 0.001

*Abbreviations*: *IQR* Interquartile range, *PE* Pulmonary embolism, *COPD* Chronic obstructive pulmonary disease, *APACHE II* Acute Physiology, Age, Chronic Health Evaluation II, *SOFA* Sequential Organ Failure Assessment, S_cv_O_2_, central venous oxygen saturation, *Hgb* Hemoglobin, *CVVH* Continuous venous-venous hemofiltration, *ECMO* Extracorporeal membrane oxygenation, *PiCCO* Pulse contour cardiac output, *PEEP* Positive end-expiration pressure, *P*_*cv-a*_*CO*_*2*_ Central venous-arterial carbon dioxide difference, *P*_*cv − a*_*CO*_*2*_*/C*_*a − cv*_*O*_*2*_ *ratio* central venous-arterial CO_2_ to arterial-venous O_2_ content difference ratio

^a^ Exposure was defined as a sustained negative central venous to arterial lactate difference during the first 24 h after admission

^b^ Major bleeding was defined as any diagnoses including bleeding and hemorrhagic shock

^c^ Cardiac dysfunction included old myocardial infarction, pericarditis, cardiomyopathy, and valvular diseases

^d^ Detailed categories of surgeries were shown in Additional file 1: Appendix Table 4

^e^ 24-hour arterial lactate clearance was defined as 100 (initial - last lactate in first 24 h)/initial lactate

**Fig. 2:**
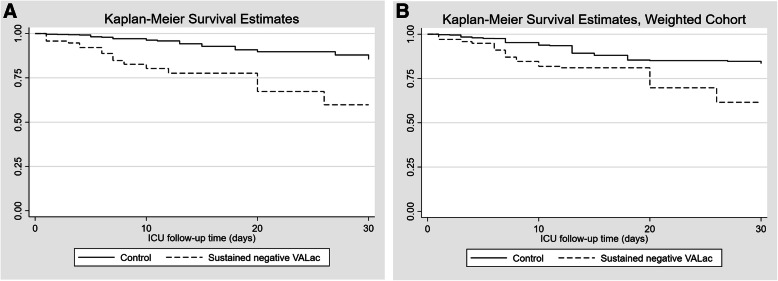
Kaplan-Meier plots for ICU 30-day mortality. **a**: unadjusted cohort. **b**: propensity score-weighted cohort (matching weight)

For the primary analysis, the patient who had sustained lower central venous lactate than arterial lactate (the exposure group) had a higher risk of death (Log-rank test *p* < 0.001), as the Kaplan-Meier plot showed in Fig. 2a. The result was consistent with the PS- weighted analysis (Fig. 2b). The SMDs after weighting showed good balance between groups (Additional file 1: Appendix Figure 1). The results from Cox proportional hazard models were shown in Table 2. After adjusted for all covariates in the multivariable Cox model, the adjusted HR was 2.31 (*p* = 0.03), which was consistent with the PS-weighted HR (HR = 2.57, *p* = 0.010). The factors that associated with the primary outcome were shown in Additional file 1: Appendix Table 1. The analysis using all VALac within the first 24 h were negative as exposure showed the higher HR than the primary analysis (HR = 2.86, *p* = 0.013), as expected. The potential factors associated with our primary exposure factor were listed in Table 3 (for multivariable logistic model) and Additional file 1: Appendix Table 2 (for univariable logistic models). Arterial lactate in the first blood gas pair, 24-h arterial lactate clearance, use of epinephrine, mean PEEP level within first 24 h, and extracorporeal membrane oxygenation (ECMO) initiation within the first 24 h showed statistically significant association with our primary exposure factor in the multivariable model.

**Table 2:** Results from Cox proportional models

Analysis^a^	Hazard ratio	95% CI	***P*** value
Unadjusted analysis	5.29	3.03, 9.24	< 0.001
Primary analysis	2.31	1.07, 4.99	0.032
PS-weighting analysis	2.57	1.17, 5.64	0.010
Use all VALac within the first 24 h were negative as exposure	2.86	1.25, 6.54	0.013

^a^: For PS-weighting analysis, *N* = 252. For other analysis, *N* = 1582

*Abbreviation*: *CI* Confidence interval, *VALac* Central venous to arterial lactate level difference, *PS* Propensity score

**Table 3:** Factors that associated with sustained negative VALac in multivariable logistic regression

Factor	Odds ratio	95% CI	***P*** value
Arterial lactate in first paired blood gas	1.24	1.16, 1.31	< 0.001
24-h lactate clearance (per 10 percentage points change)	0.97	0.95, 0.99	0.016
Epinephrine use	0.36	0.19, 0.70	0.002
ECMO initiation	6.15	1.30, 29.1	0.022
Mean PEEP level	1.16	1.03, 1.31	0.016

*Abbreviation*: *VALac* Central venous to arterial lactate level difference, *CI* Confidence interval, *ECMO* Extracorporeal membranous oxygenation, *PEEP* Positive end expiratory pressure

The results of the sensitivity analyses were shown in Additional file 1: Appendix Table 3. The association between 30-day ICU mortality and the sustained lower central venous lactate than arterial lactate was robust across all sensitivity analyses.

## Discussion

Lactate has long been used for diagnosing and monitoring tissue hypoxia, and predicting outcomes in critically ill patients [1–5]. However, data suggesting the association between the lactate level discrepancy among different sites and patient outcomes are limited. To our knowledge, our study is the first clinical medical records data analysis to evaluate the association between the central venous (SVC in our study) and arterial lactate discrepancy and outcomes in patients receiving vasopressors in ICU. The main finding of our study is that a sustained negative VALac in the early stage of treatment is associated with a poor ICU outcome as measured by ICU 30-day mortality. This association is independent with the initial arterial lactate, 24-h lactate clearance, severity scores such as APACHE II or SOFA. The sustained negative VALac is associated with a higher initial arterial lactate level, lower 24-h lactate clearance, higher average PEEP level during the first 24 h, epinephrine use (negatively correlated), and ECMO initiation.

Kellum et al. [8] reported the lactate flux from injured lung in patients with acute lung injury which lead to a lower mixed venous lactate than arterial. They also showed that the patients who had a lower mixed venous than arterial lactate had a higher lactate level and a higher probability of death. The potential mechanisms of the generation of lactate in the injured lung include the accumulation and activation of inflammatory cells in the lung, increased activity of the type II pneumocytes, and inhibition of pyruvate dehydrogenase by endotoxin, etc. [16–18]. In our study, higher PEEP level was significantly correlated with a sustained negative VALac in both univariable and multivariable models, which suggested the impacts of the severity of lung injury. Our results are consistent with the findings in previous literature [8, 16, 17] that the injured lung may be an important source of arterial lactate. It should also be noticed that the use of epinephrine was negatively correlated with the sustained negative VALac status. In septic shock patients, arterial lactate concentrations at early stage were significantly higher in patients given epinephrine only, compared with those given norepinephrine plus dobutamine, as shown in the CATS trial [19]. Johnson et al. [20] showed that, in the rat model, the infusion of epinephrine stimulated the conversion of pyruvate to lactate in the lung and the release of lactate from the lung, which decreased the transpulmonary gradient of the lactate. The use of epinephrine may contribute to the discrepancy of lactate levels between artery and SVC in our study. Detailed information of the duration and the dose of epinephrine use may help to further illustrate the impact of epinephrine on lactate metabolism.

It is also well acknowledged that brain, liver, kidney, heart, and skeletal muscle can use lactate as metabolic substrate under certain circumstances [21–24]. In patients with brain injury or under cardio-pulmonary resuscitation, studies showed the uptake of lactate in the brain [21, 25, 26], which led to a negative jugular to artery lactate gradient. We found that the acute brain injury was associated with the sustained negative VALac in the univariable logistic regression (OR = 2.04, *p* = 0.02) and showed a similar trend in the multivariable regression multivariable regression (adjusted OR = 2.01, *p* = 0.09). However, the lung injury or pneumonia related to brain injury may also contribute to the negative VALac, our data cannot differentiate those 2 potential reasons. Further studies specifically focusing patients with brain injury should be warranted.

The venous return from coronary sinus and inferior vena cava (IVC) will also affect the lactate gradient from SVC to the artery. Gutierrez et al. [9] showed the lactate concentration in mixed venous blood was lower than that in SVC or IVC, while the lactate concentration was lower in IVC than SVC without statistical significance. And Bagger et al. showed that in pace-induced angina pectoris, the lactate level in coronary sinus increased [27], which indicated that the ischemia of the heart may directly increase lactate concentration in artery but not in SVC. Our data may not fully explain the source of discrepancy in lactate levels and further studies should focus on the differences in lactate levels among different sites.

The importance of normalized lactate was highlighted in the recent guideline of septic shock [28], and lactate kinetic-guided resuscitation can reduce the mortality of patients with septic shock [29]. However, our data showed that, after adjusted for the arterial lactate of the first blood gas pair and the 24-h lactate clearance, along with other covariates, the sustained negative VALac status still showed a significant association with the 30-day ICU mortality. Our data, together with previous literature, suggest the lactate gradient between the SVC and artery can provide information that cannot be reflected by the arterial lactate only, such as brain injury, lung injury, cardiac ischemia, etc. We can infer that VALac may suggest the existence of uncorrected hypoxia or organ injury which leads to a poor outcome. Though the origin of this discrepancy has not been fully illustrated in this study, critical care physicians should pay more attention to the lactate discrepancy between different sites. Further study should focus on the treatment response of the VALac.

In spite of the differences in baseline characteristics between groups, the PS-weighting analysis and the sensitivity analyses showed robust results in concordance with the primary analysis. The propensity score-based methods have been widely used as an important statistical tool for controlling confounding in observational studies [30]. The estimate of matching weight approach is asymptotically equivalent to that of exact 1:1 matching on the propensity score, which means there is an expectation about clinical equipoise between groups and has proven to be more efficient and has better statistical properties than the pairwise matching approach [13, 14, 31]. This is in particular important for our analysis since our sample size is small. Our approach successfully balanced the baseline characteristics in the weighted cohort as measured by the SMDs. Therefore, our analysis provided a robust estimation of the association between the sustained negative VALac and the 30 day ICU mortality in our study population.

### Limitations

Our analysis has several limitations. First, this is an observational study from a single center and is subject to confounding and bias. We did not have detailed pre-ICU treatment information, which will cause bias. We included and balanced the treatment within the first 24 h after ICU admission as covariates, using them as the proxies for the unmeasured pre-ICU information. However, residual confounding related to pre-ICU treatment may still exist. Second, the performance bias would occur in our study when clinicians might want to deliver more care to sicker patients. This systemic difference in treatment performance will bias the study toward the null hypothesis. We used the number of blood gas pairs as the proxy (Additional file 1: Appendix Table 5), assuming that more paired blood gas tests ordered may suggest more intense treatment. The potential residual bias may lead to an underestimation of our result. Third, the fluctuation of lactate level matters when defining the status of continuance. Though our approach is more comprehensive than those used in some studies that only take the initial and final measures into account [32, 33], we still cannot rule out misclassification. In our analysis using “all VALac within the first 24 hours were negative” as exposure, the adjusted HR was higher than that of the primary analysis, which indicated a relationship between the mortality and the intensity of exposure. This result supported our research hypothesis. Fourth, we did not measure lactates from different sites other than SVC and artery. Therefore, we cannot specifically identify the origin of the discrepancy and the impact of underlying disease on the discrepancy. Fifth, though the mean APACHE-II score in our study cohort is in concordance with the national survey in ICU in China reported by Du et al. [34], in our study, the mortality rate was lower, along with a higher proportion of post-operation patients. And we are unable to do the disease-specific sub-group analysis due to the limited sample size. Therefore, it should be with caution to extrapolate the result of this study to ICU patients with different underlying diseases. Further studies focusing on the specific disease and the difference of lactate from multiple sites should be required.

## Conclusions

Our exploratory analysis showed that the sustained negative VALac in the early stage of treatment is associated with the poor outcome of patients receiving vasopressors in ICU and this association is independent with initial arterial lactate level or 24-h lactate clearance. Central venous lactate should not be used as a surrogate for arterial lactate. This discrepancy in lactate levels between SVC and arterial may provide additional information about the organ injury or hypoxia that may not be reflected by arterial lactate only. Critical care physicians should also pay more attention to the discrepancy of lactate levels between different sites in their clinical practice.

## Supplementary Information

**Additional file 1: Appendix Table 1.** Factors that associated (with statistical significance) with primary outcome in multivariable Cox model. **Appendix Table 2.** Potential factors that associated with sustained negative VALac in univariable logistic regression models. **Appendix Table 3.** Results of sensitivity analyses. **Appendix table 4.** Categories of surgeries received by patients in the study. **Appendix table 5.** Numbers of blood gas pairs by different factors. **Appendix Figure 1.** Standardized mean differences of each variable between exposure and control groups before and after weighting. Vertical lines indicate the goodness of balancing (between − 0.1 and 0.1).

## Data Availability

All data used and analyzed during the current study are available from the corresponding author on reasonable request.

## Abbreviations

VALac: Venous to arterial lactate gradient
ICU: Intensive care unit
HR: Hazard ratio
CCIS: Critical Care Information System
SVC: Superior vena cava
IQR: Interquartile range
CI: Confidence interval
SMD: Standard mean difference
OR: Odds ratio
STROBE: Strengthening the reporting of observational studies in epidemiology
PEEP: Positive end-expiratory pressure
ECMO: Extracorporeal membrane oxygenation
IVC: Inferior vena cava
