# Supplementary material for: Negative central venous to arterial lactate gradient in patients receiving vasopressors is associated with higher ICU 30-day mortality: a retrospective cohort study
Source: BMC Anesthesiol. 2021 Jan 22;21:25. doi: 10.1186/s12871-021-01237-5 (PMC7821722; doi:10.1186/s12871-021-01237-5)

**Negative central venous to arterial lactate gradient in patients receiving vasopressors is associated with higher ICU 30-day mortality: a retrospective cohort study**

**Supplementary material**

**List of covariates:**

Patients’ information and diagnoses were obtained from the electronic medical record at both admission and discharge. Demographic characteristics included patients’ age and gender. History of malignancy, hypertension, diabetes, coronary heart disease, cerebral vascular disease, and underlying chronic organ dysfunction were selected as baseline comorbidities. Acute scenarios at ICU admission included brain injury, infection, trauma or major bleeding, and post-anesthesia. APACHE II (Acute Physiology, Age, Chronic Health Evaluation II) scores and SOFA (Sequential Organ Failure Assessment) total scores were used as severity scores. Treatment information during the first 24 hours included arterial lactate and central venous oxygen saturation (ScvO2) from the first blood gas pair, 24-hour arterial lactate clearance [defined as 100 (initial - last lactate in first 24 hours)/initial lactate], total fluid balance, transfusion, albumin infusion, the use of vasoactive agents, mean positive end-expiration pressure (PEEP), mean oxygen index, initiation of continuous venous-venous hemofiltration (CVVH), extracorporeal membrane oxygenation (ECMO) support, and Pulse contour cardiac output (PiCCO) monitoring.

**Appendix Table 1. Factors that associated (with statistical significance) with primary outcome in multivariable Cox model*.**

| Variables | Hazard Ratio | 95% CI | P value |
| --- | --- | --- | --- |
| Sustained negative VALac | 2.31 | 1.07, 4.99 | 0.03 |
| Age | 1.04 | 1.01, 1.06 | 0.001 |
| Hypertension | 0.12 | 0.02, 0.94 | 0.04 |
| Coronary heart disease | 0.38 | 0.15, 0.96 | 0.04 |
| Cardiac dysfunction | 0.38 | 0.15, 0.95 | 0.04 |
| APACHE II score | 1.05 | 1.01, 1.10 | 0.02 |
| SOFA score | 1.18 | 1.03, 1.34 | 0.02 |
| Arterial lactate in first paired blood gas | 1.14 | 1.06, 1.23 | 0.001 |
| Dobutamine use | 3.33 | 1.19, 9.31 | 0.02 |
| Total fluid balance (in quintiles) |  |  |  |
| 1st | Reference | Reference | Reference |
| 2nd | 1.21 | 0.50, 2.88 | 0.67 |
| 3rd | 0.91 | 0.31, 2.65 | 0.86 |
| 4th | 0.17 | 0.04, 0.82 | 0.03 |
| 5th | 1.426 | 0.66, 3.07 | 0.39 |
| 24-hour lactate clearance (per 10 percentage point change) | 0.96 | 0.94, 0.99 | 0.005 |
| Number of pairs of blood gas (in quintiles) |  |  |  |
| 1st | Reference | Reference | Reference |
| 2nd | 0.68 | 0.29, 1.59 | 0.37 |
| 3rd | 0.20 | 0.05, 0.87 | 0.03 |
| 4th | 0.48 | 0.18, 1.27 | 0.14 |
| 5th | 0.30 | 0.09, 0.94 | 0.04 |

Abbreviation: CI, confidence interval; BG, Blood gas; VALac, central venous to arterial lactate gradient; APACHE II, Acute Physiology, Age, Chronic Health Evaluation II; SOFA, Sequential Organ Failure Assessment;

*: The global proportional hazard test showed a chi-square of 18.23, p= 0.92.

**Appendix Table 2. Potential factors that associated with sustained negative VALac in univariable logistic regression models**

| Variables | Univariable |  |
| --- | --- | --- |
|  | OR [95% CI] | P value |
| Malignancy | 0.66 [0.45, 0.98] | 0.04 |
| Hypertension | 0.59 [0.38, 0.91] | 0.02 |
| Cirrhosis | 3.12 [1.13, 8.65] | 0.03 |
| Acute brain injury | 2.04 [1.10, 3.81] | 0.02 |
| Serum creatinine† | 1.16 [1.05, 1.28] | 0.003 |
| APACHEII score | 1.08 [1.06, 1.10] | <0.001 |
| SOFA total score | 1.22 [1.15, 1.28] | <0.001 |
| Arterial lactate in first paired blood gas | 1.24 [1.19, 1.29] | <0.001 |
| 24-hour lactate clearance (per 10 percentage points change) | 0.98 [0.96, 0.99] | 0.008 |
| Total fluid balance (per 100ml) | 1.02 [1.01, 1.03] | <0.001 |
| Receive transfusion | 1.83 [1.26, 2.67] | 0.002 |
| Receive albumin | 1.43 [0.98, 2.08] | 0.061 |
| Epinephrine use | 1.59 [1.10, 2.29] | 0.01 |
| Milrinone use | 2.14 [1.31,3.51] | 0.002 |
| Dobutamine use | 2.80 [1.51, 5.19] | 0.001 |
| CVVH initiation | 4.08 [2.76, 6.04] | <0.001 |
| ECMO initiation | 16.5 [5.31, 51.0] | <0.001 |
| PICCO initiation | 3.65 [2.46, 5.42] | <0.001 |
| Mean oxygen index |  |  |
| >300 mmHg | Reference |  |
| 200~300 mmHg | 1.00 [0.66, 1.50] | 0.99 |
| 100~200 mmHg | 2.50 [1.58, 3.94] | <0.001 |
| ≤100 mmHg | 14.0 [4.18, 47.1] | <0.001 |
| Mean PEEP level | 1.19 [1.29, 1.39] | <0.001 |
| Number of pairs of BG | 1.26 [1.18, 1.35] | <0.001 |

†: Serum creatinine was measured in mg/dl (1mg/dl=88.4μmol/L) in this table only.

Abbreviations: APACHE II, Acute Physiology, Age, Chronic Health Evaluation II; SOFA, Sequential Organ Failure Assessment; CVVH, continuous venous-venous hemofiltration; ECMO, extracorporeal membrane oxygenation; PiCCO, pulse contour cardiac output; PEEP, positive end-expiration pressure; OR, odds ratio; CI, confidence interval; BG, Blood gas;

**Appendix Table 3.** **Results of sensitivity analyses**

| **Analysis** | **Unadjusted HR** | **95% CI** | **P value** | **Adjusted HR*** | **95% CI** | **P value** |
| --- | --- | --- | --- | --- | --- | --- |
| Primary analysis | 5.29 | 3.03, 9.24 | <0.001 | 2.31 | 1.07, 4.99 | 0.032 |
| Analysis 1 | 5.13 | 2.95, 8.89 | <0.001 | 2.79 | 1.31, 5.99 | 0.008 |
| Analysis 2 | 4.95 | 2.75, 8.91 | <0.001 | 2.91 | 1.16, 7.28 | 0.023 |
| Analysis 3 | 6.23 | 3.41, 11.4 | <0.001 | 2.97 | 1.14, 7.76 | 0.026 |

*: Adjusted for baseline characteristics and treatment within 24 hours after admission. All sensitivity analyses used a same set of covariates.

Analysis 1: Use all-cause ICU mortality as outcome.

Analysis 2: Include patients whose first blood gas pair was obtained within 4 hours after admission.

Analysis 3: Include patients who had at least 4 pairs of blood gas test obtained

Abbreviation: HR, hazard ratio; CI, confidence interval; VALac, central venous to arterial lactate level difference

**Appendix table 4. Categories of surgeries received by patients in the study**

|  | Control (1435) | Exposure* (147) | P value |
| --- | --- | --- | --- |
| Abdomen/Pelvic surgery | 505 (35.1%) | 42 (28.6%) | 0.11 |
| Thoracic surgery | 68 (4.7%) | 6 (4.1%) | 0.75 |
| Neurosurgery | 30 (2.1%) | 12 (8.2%) | <0.001 |
| Cardiac surgery | 252 (17.5%) | 28 (19.0%) | 0.64 |
| Other surgery | 128 (8.9%) | 15 (10.2%) | 0.58 |

*: Exposure was defined as a sustained negative central venous to arterial lactate difference during the first 24 hours after admission.

**Appendix table 5. Numbers of blood gas pairs by different factors.**

| Factor | In patients without factor* | In patients with factor* | P value |
| --- | --- | --- | --- |
| Post anesthesia | 5 [4,7] | 5 [4,7] | 0.72 |
| Infection | 5 [4,7] | 5 [4,7] | 0.24 |
| Cardiac dysfunction^⁋^ | 5 [3,6] | 7 [5,9] | <0.001 |
| Brain injury | 5 [4,7] | 6 [4,7] | 0.41 |
| Trauma/major bleeding^§^ | 5 [4,7] | 5 [4,6] | 0.83 |
| Mean PEEP level>5mmHg during first 24 hours | 5 [3,6] | 6 [5,8] | <0.001 |

*: Data were shown in Median [IQR]

§: Major bleeding was defined as any diagnoses including bleeding and hemorrhagic shock.

⁋: Cardiac dysfunction included old myocardial infarction, pericarditis, cardiomyopathy, and valvular diseases.

**Appendix Figure 1. Standardized mean differences of each variable between exposure and control groups before and after weighting. Vertical lines indicate the goodness of balancing (between -0.1 and 0.1)**


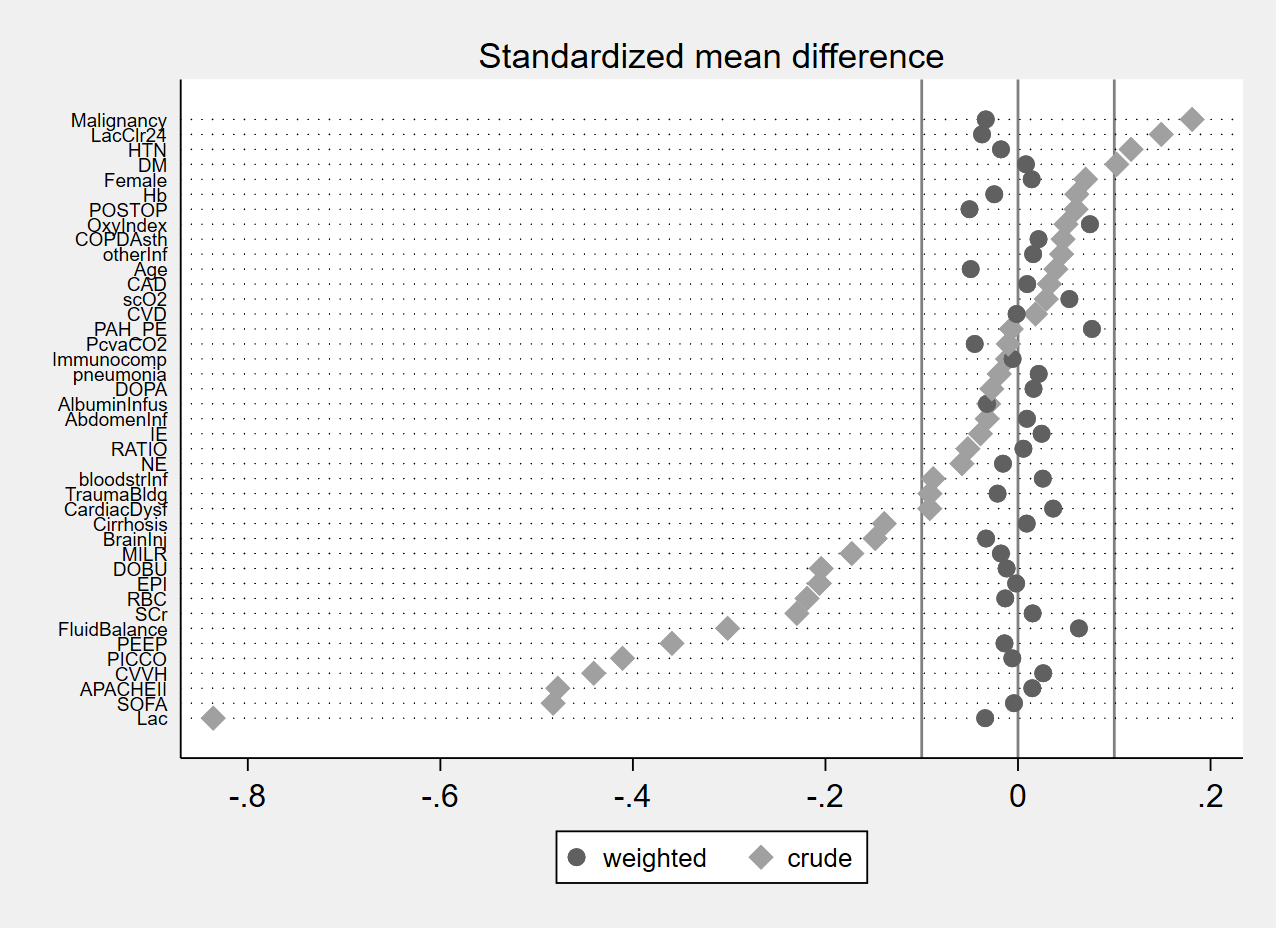

Supplement: Supplementary file 1 — Additional file 1: Appendix Table 1. Factors that associated (with statistical significance) with primary outcome in multivariable Cox model. Appendix Table 2. Potential factors that associated with sustained negative VALac in univariable logistic regression models. Appendix Table 3. Results of sensitivity analyses. Appendix table 4. Categories of surgeries received by patients in the study. Appendix table 5. Numbers of blood gas pairs by different factors. Appendix Figure 1. Standardized mean differences of each variable between exposure and control groups before and after weighting. Vertical lines indicate the goodness of balancing (between − 0.1 and 0.1). [file 12871_2021_1237_MOESM1_ESM.docx]
